# Precision in pediatric epilepsy

**DOI:** 10.12688/f1000research.16494.1

**Published:** 2019-02-06

**Authors:** Priya Sharma, Ammar Hussain, Robert Greenwood

**Affiliations:** 1 Department of Neurology, University of North Carolina School of Medicine, Physicians Office Building, Chapel Hill, NC, 27599-7025, USA; 2 Department of Neurology & Pediatrics, University of North Carolina School of Medicine, 2141 Physicians Office Building, Chapel Hill, NC, 27599-7025, USA

**Keywords:** whole exome sequencing, whole genome sequencing, epilepsy gene panels, autoimmune encephalitis, metabolic testing

## Abstract

Epilepsy in infants and children is one of the most common and devastating neurological disorders. In the past, we had a limited understanding of the causes of epilepsy in pediatric patients, so we treated pediatric epilepsy according to seizure type. Now with new tools and tests, we are entering the age of precision medicine in pediatric epilepsy. In this review, we use the new etiological classification system proposed by the International League Against Epilepsy to review the advances in the diagnosis of pediatric epilepsy, describe new tools to identify seizure foci for epilepsy surgery, and define treatable epilepsy syndromes.

## Introduction

Epilepsy is one of the most common medical problems that affect infants and children. An estimated 5% of children will have a seizure ^1^. A recent meta-analysis study found that the active period prevalence of epilepsy (the number of annual new and existing cases of epilepsy) was 4.8/1000 worldwide and in the USA was 0.94/1000 for children less than 18 years of age ^2^. In the same study, the lifetime prevalence of epilepsy (the number of active and existing cases of epilepsy between birth and the time of assessment) for children less than 18 years of age was 7.2/1000 ^2^. Epilepsy and the conditions that cause epilepsy impact children and their families in many different ways, impacting cognition, behavior, and socioeconomic status. Uncontrolled epilepsy and the conditions that cause seizures leave indelible changes that can affect a child for life and even increase the risk of sudden death. We have had a limited understanding of the pathophysiology of epilepsy and limited tools to diagnose epilepsy and its cause. In recent years, however, the diagnosis of the cause of epilepsy in infants and children has dramatically improved. The classification of epilepsy has changed; we no longer use the same terminology to describe seizures and epilepsy. New methods to identify the cause of seizures and epilepsy are now available; as a result, we have found new ways to treat seizures and epilepsy. We have new tools to distinguish types of epilepsy and to localize the seizure focus in focal epilepsy. With a better understanding of epilepsy pathophysiology and new ways to diagnose and treat epilepsy, the International League Against Epilepsy (ILAE) has developed a new classification system ^3^. Epilepsy in infants and children has entered the age of precision medicine. We are closing in on the goal to identify and treat the cause of epilepsy rather than the symptoms. How has our ability to identify an epilepsy etiology changed? What new tests are available for making diagnoses in each of the etiological categories in the ILAE classification? How do you use these tools? This review will answer some of these questions.

## Changes in epilepsy classification

**Figure 1.**
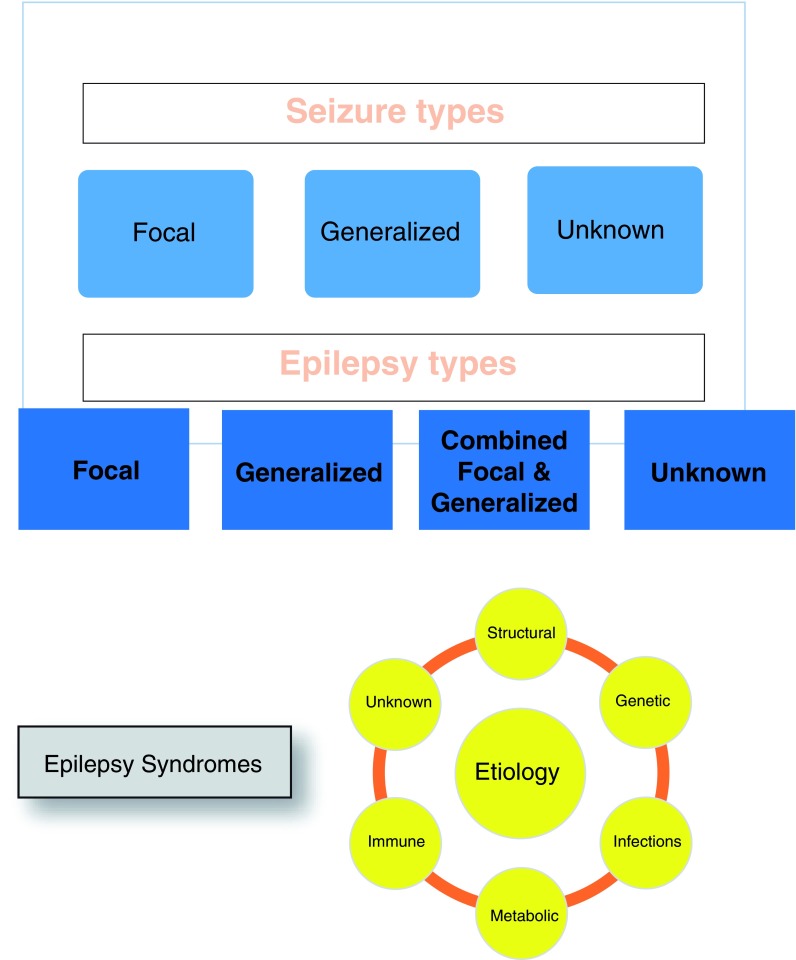
Classification of seizures and epilepsy. Epilepsy: At least two unprovoked (or reflex) seizures occurring more than 24 hours apart or one unprovoked or reflex seizure and an at least 60% chance of recurrent seizures over the next 10 years or the diagnosis of an epilepsy syndrome.

The ILAE recently reclassified seizures and epilepsies. The classification still depends primarily on clinical and electroencephalography (EEG) features but with modest changes in some of the terminology ^3^. The term *focal onset* replaces the term *partial onset*, and *focal onset* seizures are placed into two categories: *aware* and *impaired awareness*. Focal seizures that become generalized seizures are now referred to as *focal to bilateral tonic-clonic* instead of partial with secondary generalization. Many of the descriptions of the motor and non-motor activity that occurs during the seizure remain the same. The most significant change has been the move toward classifying epilepsies according to epilepsy etiology and a better definition of epilepsy syndromes. Figure 1 lists the new classification system and the six etiological categories in the ILAE classification. This change recognizes some of the important advances in defining etiologies for epilepsy, particularly in children and infants with seizures.

The etiological classification has six categories ( Figure 1). As Falco-Walter *et al*. noted in describing the changes in the ILAE classification of seizures and epilepsy, “from first contact with the patient, the clinician is encouraged to consider the etiology of their seizures” ^3^. This is an important move, as epileptologists have long known that establishing a specific etiology was important for optimizing treatment and that defining epilepsy syndromes was an important first step in the process of defining specific etiologies and treatments. The advances in basic and clinical sciences have provided many new diagnostic avenues and tools that allow even greater diagnostic and therapeutic precision. This can be compared with the importance of etiology in infectious disease.

## Epilepsy diagnosis

A recent population-based study from Australia provides important new data regarding the etiology of severe epilepsies of infancy ^4^. In this study, the authors analyzed records of infants whose seizures began before age 18 months and were frequent, associated with an epileptiform EEG, and failed to respond to two or more antiepileptic drugs. The authors were able to identify 114 cases that were born during 2011–2013 and found that 14% had acquired brain insults, 54% had genetic/presumed genetic etiologies, and 33% had unknown etiologies. Of the 54% (62) that had genetic/presumed genetic etiologies, 31 had structural abnormalities (usually dysplasias or cortical migrational defects), six had metabolic etiologies, nine had chromosomal etiologies, and 16 had single-gene etiologies. This study shows the importance of the subsequent advances in diagnosis that we will describe. The study also highlights how whole exome sequencing (WES) has changed the financial value of genetic testing, increasing diagnostic yield at a lower cost.

## Precision in structural causes of epilepsy and surgical management

### Advances in imaging for structural causes of focal epilepsy

Structural abnormalities represent a well-known and prominent cause of epilepsies affecting the pediatric population. Fortunately, neurosurgical intervention can be quite successful in treating epilepsy caused by these structural abnormalities if they are properly localized. Traditional imaging sequences, however, miss many of these abnormalities, especially microstructural abnormalities. Table 1 presents a list of the recent advances in imaging and electrophysiology that have improved localizing dysplasias and epileptogenic foci in the brain.

**Table 1.** Advanced localization techniques in magnetic resonance imaging, magnetoencephalogram, and electrophysiology.

Magnetic resonance imaging (MRI)			
Technique	Description	Advantages	Limitations
Diffusion tensor imaging (DTI)	MRI diffusion technique that measures restricted diffusion of water in tissues by using a multi-directional tensor model applied to diffusion measurements within each voxel	- Has been used successfully in epilepsy surgery - Can be used for tractography	- Assumes Gaussian (normal) water movement and brain barriers restrict water movement to different degrees
Diffusion kurtosis imaging (DKI)	DKI is similar to DTI, but it follows a non-Gaussian diffusion probability distribution, also known as kurtosis. Thus, it is considered more sensitive than DTI.	- More sensitive than DTI and better at detecting epileptic focus pathology	- No studies of epilepsy surgery application
Neurite orientation dispersion and density imaging (NODDI)	NODDI produces microstructural images of neurites, which are composed of dendrites and axons, neurite dispersion, and neurite orientation *in vivo*.	- Takes into account the differences in water (proton) movement in three different compartments and the differences in different brain structures (for example, corpus callosum versus gray matter)	- Requires large voxels in comparison with structural imaging unless stronger imaging gradients are used - No studies of epilepsy surgery application
Other imaging techniques: magnetic source imaging			
Magnetoencephalogram (MEG)	Acquires high spatio-temporal resolution using current dipole modeling of interictal spikes and localization of estimated dipole onto the patient’s own MRI	- Can localize epileptic spikes even from deep foci - Can be used with statistical parametric mapping - Successful use in epilepsy surgery	MEG facilities are very expensive, so MEG studies are expensive. - Difficult to use to capture a seizure
Electrophysiological techniques: Electrical source imaging is a model-based approach for imaging electrical sources associated with brain activation from multi-electrode non-invasive electroencephalography (EEG).			
Stereoelectroencephalography (SEEG)	Implantation of intracerebral multi-contact electrodes under stereotactic guidance for intracranially recording epileptiform activity, access to deep cortical structures, analyze spatiotemporal organization of epileptogenic networks, and localize the epileptogenic zone	- Used with statistical parametric mapping - Can localize epileptic zone as accurately as MEG and is less expensive	Invasive since implantation of electrodes is required
High-density electroencephalogram (HD-EEG)	Enhances spatial resolution of EEG by utilization of up to 256 electrodes analyzing data with source- localization algorithms	- Can be applied to ictal and interictal recordings and electrodes are extracranial - Localization superior to ictal single-photon emission computed tomography (SPECT), positron emission tomography, and conventional EEG	Requires special software
HD-EEG fast functional magnetic resonance imaging (fMRI) (magnetic resonance encephalography)	Combines the high temporal resolution of HD-EEG with the high spatial resolution of fast fMRI aiming at localization of epileptogenic zone based on the associated blood oxygenation level-dependent (BOLD) response	- Can be applied to interictal recordings - May identify a focus missed by other techniques	- Fast fMRI may lead to more false- positive activations from noise. - Focus localization is difficult because more parts of a network are identified. - The short duration of a fast fMRI study may be negative because of only a few spikes.

This table lists the most recent advances in imaging and electrophysiology that have improved the identification of brain malformations and epileptic foci. It lists procedures that are commonly used and others that epileptologists have only begun to use.

The addition of voxel-based morphometry (VBM) to magnetic resonance imaging (MRI) processing and analysis can significantly overcome this obstacle and enhance microstructural changes. A prime example can be seen in focal cortical dysplasia (FCD), a complex and highly epileptogenic malformation. The addition of VBM to traditional MRI images is consistently more accurate in measuring cortical thickness and gray-white contrast, and its other measurements (for example, sulcal depth/gyrification and Jacobian distortion) result in a more exact illustration of FCD ^5, 6^. Complete surgical resection can successfully alleviate epilepsy burden, but traditional MRI analysis fails to capture FCD in more than half of patients with histological confirmation ^7^.

Diffusion tensor imaging (DTI), diffusion kurtosis imaging (DKI), and neurite orientation dispersion and density imaging (NODDI) represent advanced sequences that produce more sensitive pictures compared with T1 and T2 ^8^. They also enhance the diagnostic advantages displayed by VBM and tractography, whether in addition to or in place of traditional MRI sequences ^5, 8– 12^. This has been seen in temporal lobe epilepsy (TLE) and in FCD, where DTI-based VBM analysis assisted in accurate FCD subtype classification ^8, 12^. In children diagnosed with active idiopathic epilepsy with and without cognitive impairment, DTI-based VBM and tractography analysis showed subtle microstructure and connectivity patterns that correlate to cognitive impairments (for example, verbal and memory) with relation to age of first seizure ^11^. DKI and NODDI are even more sensitive than DTI ^8^, but currently their use in pediatric epilepsy is limited. Research into their applicability is warranted.

### Advances in defining the epileptogenic zone in focal epilepsy

Advances in imaging techniques, such as simultaneous EEG-functional magnetic resonance imaging (EEG-fMRI) and high-density EEG (HD-EEG), have been notably helpful in pre-surgical planning. The introduction of simultaneous EEG-fMRI monitoring has made more precise and accurate determination of epileptic electrical structural correlates more feasible ^13^, leading to more successful neurosurgical treatments. Work done on expanding its measuring capabilities, such as spatiotemporal mapping ^14^, can provide even more relevant epileptogenic details. HD-EEG is another technique recently shown to reliably predict complete resection of epileptogenic networks. In a recent study ^15^ exploring different pre-surgical imaging tools—that is, MRI, positron emission tomography, single-photon emission computed tomography (SPECT), HD-EEG, and a combination of devices—and their associated post-operative seizure freedom rates, HD-EEG was the most sensitive by a large margin but was limited in its specificity (87.8% versus 47.1%, respectively). Specificity improved when HD-EEG was combined with another imaging technique, most notably MRI, but was not as sensitive (88.2% versus 58.5%, respectively) ^15^.

Magnetoencephalography (MEG) is another non-invasive technique that stands out from the two previously mentioned techniques because of the high spatial and temporal resolution it provides ^16^. MEG improves epileptic foci associated with cortical dysplasia and surgical outcome. In a recent study ^16^, MEG localization showed a 91% capture rate for histopathically confirmed FCD (82% of type I and 100% of types II and III), compared with a 64% capture rate with MRI (59% of type I, 62% of type II, and 100% of type III). As MEG has the ability to pick up electrical activity in deep brain structures, it has been more accurate than non-invasive video EEG monitoring in mapping epileptogenic zones; more specifically, in localizing seizure foci, the high sensitivity of MEG for localizing seizure foci remains consistent even in the infant population ^17^. The advances in quantitative algorithms to model the epileptogenic zone and growing popularity of stereoelectroencephalography (SEEG) allowing exploration of deep epileptogenic foci, performing feasible bilateral intracranial recordings, and three-dimensional mapping of epileptogenic zones have opened novel surgical treatment options. Utilization of SEEG in detection and algorithmic analysis of high-frequency oscillations in a recent study showed an average sensitivity of 81.94% and an average specificity of 96.03% in identification of seizure onset ^18^. Current available data indicate that utilization of SEEG enhances the chances of underlying epilepsy surgery with seizure-free outcome rates ranging from 50 to 88% ^18^. Overall, these imaging advances with improved electroencephalography more precisely detect epileptogenic zones and foci, which in turn directly improve surgical treatment outcome.

## Advances in precision medicine: advances in metabolic and genetic testing

Neurology and genetics have made amazing progress in identifying the etiology of epilepsy in infants and children. We will describe some of the changes in metabolic and genetic testing and provide some guidelines for their use.

## Metabolic tests

Metabolic testing for diseases that cause epilepsy has greatly expanded in the last few years, and new methods for testing many metabolites are now available. Table 2 provides a list of the metabolic tests for diseases that cause epilepsy in pediatric patients. Many of the tests listed in the table are panels organized by the type of metabolic disease. These panels include tests for different metabolic diseases in the same metabolic pathway. Different medical institutions and companies provide these tests. An internet search using the terms in Table 2 will provide a list of the companies and institutions that offer the tests, the sample requirements, the cost of the test, and how to send the samples. The metabolic tests in Table 2 are only screening tests, and confirmation of a disease would require additional testing. Interpretation of the tests may require help from an expert in metabolic diseases. The list is very extensive but the choice of tests usually can be reduced by considering the patient’s clinical and laboratory features.

**Table 2.** Metabolic tests for pediatric epilepsy.

Blood	Disorder tested
Chem 20	Diseases causing low Na, Ca, Mg, or Glu or liver or renal diseases
Amino acids	Aminoacidurias
Acylcarnitine profile, total and free carnitine	Mitochondrial disorders, fatty acid oxidation defects, and ketone body metabolic disorders
Lactate	Defects in energy metabolism
Pruvate	Defects in energy metabolism
Lysosomal enzymes	Lysosomal storage diseases
Peroxisomal fatty acid panel	Peroxisomal disorders
Glucose-deficient transferrin assay (isoelectric focusing of serum transferrin)	Glycosylation disorders
Homocysteine	Homocysteinuria
Pipecolic acid	Some peroxisomal disorders, mitochondrial disorders
Biotinidase activity	Biotinidase deficiency
Urine	
Organic acids	Organic acidurias
Amino acids	Aminoacidurias
Sialic acid	Sialin deficiency (Salla disease)
Alpha amionadipic semialdehyde	Pyridoxine-responsive seizures and folinic acid-responsive seizures Molybdenum cofactor deficiency
Creatine and guanidinoacetate	Cerebral creatine deficiency syndromes, creatine deficiency syndromes
Glycosaminoglycans (GAGs)	Mucopolysaccaharidoses
Oligosaccarides	Oligosaccahridoses; some gangliosidoses
Purine and pyrimidine panel	Hypermethionemia due to adenosine kinase deficiency, dihydropyrimidine dehydrogenase deficiency, dihydropyrimidine deficiency, beta-ureidopropionase deficiency
Sulfocysteine	Molybdenum cofactor deficiency, sulfocysteinuria
Cerebrospinal fluid	
Protein	Elevated in many severe epilepsies
Glucose (match with serum glucose)	Glucose transporter defect (cerebral folate deficiency)
Neurotransmitter metabolites	Metabolites Neurotransmitter metabolites are elevated in at least eight different diseases, some of which cause seizures.
Tetrahydromethylfolate	Cerebral folate deficiency
Sialic acid	Sialin deficiency (Salla disease)
Amino acids	Non-ketotic hyperglycemia, glycine encephalopathy, serine biosynthesis defects, sulfite oxidase deficiency, and others
Lactate	Defects in energy metabolism
Pyruvate	Defects in energy metabolism
Pyridoxal 5′-phophate	Hyperprolinemia type 2, alpha aminoadipic semialdehyde dehydrogenase deficiency, pyridoxine phosphate oxidase
Neopterin/Tetrahydrobiopterin	Immune system marker
Alpha aminoadipic semialdehyde	Pyridoxine-responsive seizures and folinic acid-responsive seizures
Succinyladenosine	AICA-ribosiduria
4-hydroxybutyric acid	Succinic semialdehyde deficiency
Folate receptor antibody	Cerebral folate deficiency
Other samples	
Fibroblasts	Storage diseases, Sjögren–Larsson syndrome, other specific enzyme tests
Bone marrow biopsy	Niemann–Pick type C; neuraminidase deficiency
Hair	Heavy metals
Leukocytes	Storage diseases, Sjögren–Larsson syndrome, other specific enzyme tests
Muscle biopsy	Mitochondrial diseases

This table lists individuals and panels for metabolic tests for metabolic disorders that cause epilepsy. It lists what samples are needed and what metabolic disease or pathway is tested. Clinical features of a pediatric patient with epilepsy should dictate the choice of tests.

The tests in Table 2 are biochemical tests for specific diseases in specific pathways. Metabolomics/metabonomics use “quantitative measurement of dynamic multiparametrics metabolic response of living systems to pathophysiological stimuli or genetic modification” ^19^, an approach pioneered by Nicholson *et al*. ^20^. Metabolomics analyzes the metabolome, organic substances occurring from metabolism in the body, and xenobiotics, foreign substances that are not naturally found in an organism. Metabolomics is closer to the phenotype of an organism than genomics, transcriptomics, or proteomics. Metabolomics complements genomics. Metabolomics is a relatively new method that analyzes plasma, urine, saliva, cerebrospinal fluid, and other samples by using mass spectrometry (metabonomics) or nuclear magnetic resonance spectroscopy (metabonomics) or both to quantify, for clinical diagnostic purposes, many metabolites. This testing is only for small molecules, so metabolomic testing does not test for large-molecule diseases like lysosomal storage diseases, mucopolysaccharidosis, mucolipidosis, or congenital disorders of glycosylation. Metabolomic testing is available through several laboratories. Interpretation of the results requires a skilled biochemist. Currently, we do not know with certainty whether metabolomics will play a larger role in the diagnoses of diseases causing epilepsy in pediatric patients, but the field is rapidly growing and has the same attraction as WES or whole genome sequencing (WGS) does. Currently, metabolomics is used when other diagnostic testing has failed to identify an etiology and to confirm an abnormal finding in metabolic testing or to help determine whether a variant of unknown significance in genetic testing is significant.

## Genetic testing

Genetic abnormalities reportedly cause or influence more than 70% of epileptic conditions (that is, conditions in which epilepsy presents as a core or associated symptom) ^21^. Determining whether a gene mutation causes epilepsy is complicated because single-gene mutations can cause variable phenotypes and different gene mutations cause the same phenotype. As genetic epilepsy syndromes tend to manifest in infancy or childhood, genetic testing has become an integral part of pediatric epilepsy work-ups. The evolution of diagnostic techniques due to massively parallel sequencing has allowed a rapid evolution from narrowly applicable tools (for example, fluorescence *in situ* hybridization [FISH] and single-gene testing) to multigene panels, clinical exome sequencing, clinical genome sequencing, and chromosomal microarrays for testing infants and children with epilepsy. Table 3 lists the types of genetic testing for genetic causes of epilepsy and their advantages and disadvantages.

**Table 3.** Genome sequencing tests.

Genetic tests	Method	Types and technical differences	Advantages	Disadvantages
Multigene panels	- Sequences groups of genes causing a phenotype	- Sequence analysis with/without deletion/duplication analysis	- May be able to detect mutations that are missed in comprehensive gene testing (for example, *ARX* gene and *SCN1A* ^23^) - Can design specific multigene panels - Generates fewer variants of unknown significance	- Tests only for the genes in the panel unless done as part of whole exome sequencing
Comprehensive gene testing				
Exome sequencing	- Sequences protein coding regions only	- Sequence enrichment - Single- or paired-end sequencing - Read depth - Accuracy of base calling - Family testing-trio sequencing	- More useful for hard-to-characterize epilepsy phenotypes - Sequencing has reported sensitivity. - Covers genes that may not be in multigene panels - Can identify variants of uncertain significance that may be pathogenic - Can be reanalyzed	- Generates a large number of variants of unknown significance - Cannot detect imprinting errors, uniparental heterodisomy, nucleotide repeats, pseudogenes, non-coding regions, mitochondrial genes, mosaic changes, large copy number variation, or chromosome rearrangements - Results may take four or more weeks to return.
Genome sequencing	- Sequences all coding and non-coding regions	- Has similar laboratory limitations as listed for exome sequencing	- Has the same advantages as exome sequencing - Less arduous sample preparation - Can identify structural variants and chromosome breakpoints in non-coding regions	- Many of the same limitations as exome sequencing - Some exons may not be sequenced - More expensive than exome sequencing
Chromosome microarray	- Detects copy number deletions or duplications of variable sizes	- Oligonucleotide array (comparative genomic hybridization) - Polymorphism genotypic (single- nucleotide polymorphism)	- Available in many medical facilities - Covered by insurance and Medicaid	- Does not analyze all exomes or genome - Does not sequence genes in the targeted regions analyzed

This table lists currently available sequencing methods and their advantages and disadvantages. All of these tests are commercially available and we review the yield of these tests in studies of pediatric patients with epilepsy. The information in the table is from Wallace and Bean ^24^ and Helbig *et al*. ^23^.

Next-generation sequencing (NGS) is expanding the list of epilepsy-related genes as well as uncovering variant patterns that potentially could predict pathogenicity, disease pathophysiology and evolution, and therapeutic targeting. Table 4 shows the results from studies involving pediatric patients with epilepsy. Most of the studies involve epilepsy gene panels or WES sometimes in combination with an epilepsy gene panel. Because of the expense, only one of the studies used WGS. The number of genes in the epilepsy panels is rapidly increasing, and an epilepsy panel that includes mitochondrial gene testing and that covers a total of 553 genes is now available from MNG Laboratories (Atlanta, GA, USA) ^22^. The cost of whole exome and whole genome testing is decreasing rapidly enough that these genetic tests may be the next test after imaging. As Table 4 shows, genetic testing has the highest yield in pediatric patients, especially infants with epilepsy syndromes or infant epileptic encephalopathies.

**Table 4.** Sequencing studies in pediatric epilepsy.

Phenotype	Number	NGS test (number of genes)	Diagnostic rate	Reference
EE	10 trios	WES	66%	25
EIEE	6 trios	WGS	67%	26
EE (IS, LGS)	356 trios	WES	12%	27
IS	18 trio	WES	28%	28
EE, ES	9 trios	WES	77%	29
PME	84 single	WES	31%	30
IS	10 trios	WES	40%	31
E EE	293 trio and singles	WES	38% 43%	32
EE	32 trios	WES	50%	33
EIEE	14 trio	WGS	100%	34
EIEE	14 trios	WES	36%	35
SEI	114	WES	56%	4
EIEE	733	WES	42%	36
DRE (abstract)	74	WES	17.3%	37
	141	E Panel	32.6%	37
	58	Targeted WES	44.8%	37
Focal	40 single	Targeted WES (64)	12.50%	38
Many	19	E Panel (67)	47%	39
Many	339	E Panel (110)	18%	40
E	87	E Panel (1/2 - 83, 1/2 - 106)	19.50%	41
EE	105	EE Panel (71 genes)	28.50%	42
EIEE	733	E Panel (2742 genes)	26.70%	36

This table lists the diagnostic yield in studies using whole genome sequencing (WGS), whole exome sequencing (WES) +/- gene panels, and epilepsy gene panels. It gives the number of probands and the studies using trios (sequencing the proband, mother and father). The diagnostic rate refers to the percentage of patients sequenced who had pathogenic mutations in genes known to cause epilepsy. The studies were done at different times (early sequencing did not include some of the genes we now know cause epilepsy in children) and their definition of pathogenicity varies. Only one of the studies—Howell *et al*. ^4^—can be called a population study and it was in only a subset of infants with intractable epilepsy. In general, infants with severe generalized epilepsy or epilepsy syndromes have the highest diagnostic yield with gene sequencing. DRE, drug-resistant epilepsy; E, epilepsy (included adults and children); E Panel, epilepsy gene panel; EE, epileptic encephalopathy; EIEE, early infant epileptic encephalopathy; ES, epilepsy syndromes; IS, infantile spasms; LGS, Lennox-Gastaut syndrome; PME, progressive myoclonic epilepsy; SEI, severe epilepsies of infancy.

In addition to NGS, epigenetic biomarkers are starting to play a role in enriching diagnostic precision in genetic and non-genetic pediatric epilepsies. Epigenetic factors are vast, and DNA methylation, histone modification, and non-coding RNA are some of the most impactful in pediatric epilepsy. Such factors provide a more detailed diagnostic value, as evidence of their presence can clarify disease manifestations and evolution. A prime example is the possibility of using DNA methylation as a diagnostic epigenetic biomarker in TLE, a non-genetic condition, after recent studies have shown DNA methylation to be widespread in TLE. DNA methylation as an epigenetic biomarker can also enhance disease progression and prognostic predictions for many genetic epilepsies (for example, Dravet, benign familial neonatal seizures, and epileptic neurodevelopmental disorders, or NDDs) as they regulate the associated disease and disease-modifying gene expression and functioning (for example, *KCNQ3*, *SCN3A*, and *GABRB2*, respectively).

In choosing metabolic or genetic tests, the clinician has many challenges that include deciding the sequence of testing, choosing the type of screening test, considering the cost of the tests, and comparing the quality of the laboratory tests. We have discussed some of these issues above. Confirming that a patient has epilepsy by the clinical history and an EEG must be the first step followed in most cases by imaging to uncover structural causes of epilepsy. The subsequent sequence of biochemical or genetic testing to define an etiology is changing. Finding a treatable cause of epilepsy should always accompany trying to control the seizures and in some cases control of the seizures will only be possible when you find the cause of the epilepsy. A pediatric patient with epilepsy should receive a therapeutic trial with pyridoxine, folinic acid, and biotin even if you have done biochemical and genetic testing. Table 5 lists treatment and gene mutations for some of the treatable causes of epilepsy. Early diagnosis and treatment of these disorders are essential to improve long-term outcome.

**Table 5.** Treatable epilepsies in pediatric epilepsy.

Type of epilepsy	Gene	Treatment
Cerebral folate deficiency	Folate receptor defect or folate receptor antibody	Folinic acid or methylfolate
Pyridoxine-responsive epilepsy	*ALDH7A1*/alpha aminoadipic semialdehyde	Pyridoxine and folinic acid
Pyridoxal 5′-phosphate-dependent epilepsy	*PNP0*/ *PNP0* enzyme	Pyridoxal 5′-phosphate
Glucose transporter defect	*SLC2A1*/glucose transporter protein type 1	Ketogenic diet
Biotinidase deﬁciency	BTD/biotinidase	Biotin
Biotin-thiamine-responsive basal ganglia disease	SLC19A3/thiamine transporter protein	Thiamine and biotin
Serine synthesis defects	*PHGDH*, *PSPH*, *PSAT* genes	Oral L-serine
Creatine deﬁciency syndromes	*SLC6A8*/ *GAMT*	Dietary arginine restriction and creatine- monohydrate and L ornithine supplementation
Riboﬂavin transporter deﬁciency	SLC52A2/RFVT2	Riboflavin
Molybdenum cofactor deficiency A	*MOCS1* and *MOCS2*	Purified cyclic pyranopterin monophosphate
Tuberous sclerosis	*TSC1* OR *TSC2*/hamartin	Vigabatrin mammalian target of rapamycin (mTOR) inhibitors: rapamycin, serolimus, and everolimus
POLG gene disorders	*POLG* genes	

This table provides the growing list of epilepsy for which we have specific treatment that addresses the underlying abnormality causing the seizures. Some of the treatments do not fully reverse the effects of the underlying disorder as in pyridoxine-responsive epilepsy. Many of the metabolic diseases treated by dietary changes are not listed in this table. Most of these are found with newborn testing.

The choice of testing is highly dependent on how well a pediatric patient fits an epilepsy syndrome or known genetic disorder. As noted by Myers *et al*., recognizable syndromes like tuberous sclerosis may not require genetic testing unless the testing is for genetic or prognosis ^43^. In some cases, like Rett syndrome, the symptoms and signs would help with the diagnosis but would not be enough to confirm the diagnosis. Genetic testing could be limited to testing for an *MECP2* gene mutation and, if this were negative, could be expanded to a Rett-like gene panel ^43^. In the case of Dravet syndrome, a gene panel that includes the *SCN1A* gene should be the first genetic testing given that single-gene tests may be as expensive as gene panels or more so. The cost of genetic testing sometimes may limit the choices. Interpretation of the test results when using comprehensive genomic testing can be challenging and often requires the help of a geneticist or genetic councilor or both ^23^. However, a recent cost analysis reported by Howell *et al*. ^4^ found that, in a population-based study of severe epilepsies of infancy, the early use of WES omitting some metabolic testing provided the highest diagnostic yield for the lowest cost. This study strongly suggests that the improvement in diagnostic precision in a patient with epilepsy increasingly calls for the use of gene panels, exome sequencing, or genome sequencing. The exome and genome testing improves when done as a trio, testing the parents of the patient along with the patient, but this sometimes adds cost. Several recent studies have shown that exome and genome sequencing tests significantly improve diagnosis in patients with epilepsy ^4, 43, 44^.

## Advances in immune disease testing

In the last decade, the list of autoimmune neurological diseases has rapidly expanded. Many of them cause seizures. Table 6 provides a list autoimmune encephalitides and the antigen targets for the autoantibodies that cause seizures. Autoimmunity as a cause for seizures and epilepsy should be considered in pediatric patients who were previously normal and then present with acute or subacute onset of seizures and signs of infection, additional behavioral symptoms, or movement disorders. Brain imaging abnormalities and an abnormal EEG can also support the diagnosis of presumed autoimmune encephalitis ^45, 46^. Antibody testing for autoimmune encephalitis is available, but prompt immunomodulatory treatment (corticosteroid and intravenous immunoglobulin, plasma exchange, and rituximab) and testing for paraneoplastic tumors should come before the results are available ^45, 46^. Prompt treatment can be very effective.

**Table 6.** Autoimmune encephalitis studies in pediatric epilepsy.

Surface antigens (channels and receptor targets)	
Epilepsy syndrome/epilepsy type	Autoimmune marker
Rasmussen	Glutamate receptor targets ○ AMPAR ▪ GluR3 ^47, 48^ ▪ GluR2-GluR3 Complex ^47, 49^ ○ NMDAR GluN2 ^48^
FIRES	GABA _A_R ^48, 50^ VGKV Complex ^48^ GAD65 ^48^ AMPAR ^48^ GluA3 ^48^
West syndrome	NMDR ^51^ GABA _A_R ^51^ GAD ^51^
LGS	GABA _A_R ^51^ GlyR ^51^
Human AD Lateral TLE	Contactin 2 ^46^
Severe epilepsy - status epilepticus, epilepsia partialis continua	GABA _A_R ^46, 48, 51, 52^
Encephalitis with Faciobrachial Dystonic Seizures	LGI1 & CASPR2 ^46, 48, 51, 52^
Progressive encephalomyelitis with rigidity and myoclonus	GlyR ^51, 52^
Cerebral folate deficiency Myoclonic, atypical absence, generalized tonic-clonic seizures and developmental delay and irritability	FRα ^53^
**Limbic Encephalitis** Anti-AMPAR-psychosis and seizures Anti-CASPR2-Neuromyotonia, polyneuropathy, bulbar weakness Anti-NMDAR- psychiatric changes, partial seizures, movement disorders Anti-GABA B- frequent temporal lobe-onset simple and complex partial seizures. Neuropsychological changes Focal onset Seizures memory and psychiatric changes, partial seizures, insomnia	AMPAR ○ GluA1 ^46, 48^ ○ GluA2 ^46, 48^ CASPR2 ^51^ NMDAR ○ GluN1 ^48, 51, 52^ GABA _B_R ^48, 52^ VGKC Complex ^52^ Contactin 2 ^46^ *CASPR2* LGI1 ^46, 48^
Other limbic encephalitis	GAD/GAD65 ^46, 48, 51, 52^ Ri (Nova) ^46, 48^ Yo ^46, 48, 52^ Hu ^46, 48, 52^ MA2 ^46, 52^ Tr ^46, 48, 52^ Amphiphysin ^46^ CRMP5 ^46^ DNER ^46^

This table lists many of the identified auto-antibodies that are associated with different encephalitides that cause seizures in pediatric patients. It lists antibodies according to their mechanism of action, clinical syndromes, and nervous system antigen target. An evaluation confirming possible autoimmune encephalitis and treatment for autoimmune encephalitis should precede the return of test results for autoimmune encephalitis. AMPAR, AMPA receptor; CASPR2, contactin-associated protein-like 2; FRα, folate receptor α; GABAAR, GABA A receptor; GAD, glutamic acid dehydrogenase; GAD65, Glutamate dehydrogenase protein; GluA1, GluA2, Glur2, and GluR2 and 3, AMPA receptor subunits; GluN2, NMDA subunit; GluA3, GluN2, and GluN1, NMDAR subunits; GlyR, glycine receptor; NMDR, NMDA receptor; VGKC- and LGI1, voltage-gated K channel complex proteins; VGKV complex, voltage-gated K channel protein.

## End

We have described many of the advances in testing to find the cause of epilepsy in a pediatric patient. As the use of NGS continues, so does our ability to capture epileptic significance in many genes that had either no prior seizure association or limited seizure association. A prime example is the substantiation of *GABRB2*, *SNAP25*, *CACNA1E*, and *KCNQ3* as epileptic NDD-related genes ( *KCNQ3* has prior association with benign familial neonatal seizures ^44^). This in turn will not only help tailor and increase diagnostic rates for genetic panels but also will help our understanding of the complex pathogenesis underlying genetically mediated epilepsy.

The use of the new diagnostic tools begins with a good history and physical examination and that may provide most of the information needed to find the cause of epilepsy in a child. The history and examination are also essential for choosing tests and interpreting test results. An infant with a history of a perinatal stroke confirmed by MRI imaging and clinical focal seizures, confirmed by an EEG has a clear etiology for focal onset epilepsy, a stroke, and does not require further laboratory testing to find the cause of the seizures. Many infants and children, however, do not have a history or physical examination features to allow a specific diagnosis and these are often the patients with intractable epilepsy, who (as noted above) most benefit from the advances in laboratory diagnosis. The laboratory testing that is now available has dramatically improved our ability to make specific diagnoses. The improvements have been in more and better methods for identifying metabolic abnormalities and genetic abnormalities. The explosion of new tests presents a challenge for clinicians caring for infants and children with epilepsy. Clinicians have to make difficult choices in choosing the right tests, interpreting the results, and using the information to provide earlier and better treatment. These decisions include considering the cost of tests, and at least one study suggests that in early-onset epileptic encephalopathies or drug-resistant epilepsy, genetic testing should be used early in the course of diagnosis.

Improved methods to identify structural, metabolic, genetic, and autoimmune diseases affecting the nervous system have also given us new tools to treat epilepsy. The disorders listed in Table 5 are only the start of precision treatment for epilepsy. The precise definition of the cause of epilepsies opens the door for new drugs, protein replacement, gene therapy, and many other promising treatments for the most devastating epilepsies that affect infants and children. Improvements in localization of developmental/structural changes that cause focal epilepsy are also on the horizon. We are at one of the most exciting times in epilepsy, truly a time of precision medicine.

## Abbreviations

DKI, diffusion kurtosis imaging; DTI, diffusion tensor imaging; EEG, electroencephalography; FCD, focal cortical dysplasia; fMRI, functional magnetic resonance imaging; HD-EEG, high-density electroencephalography; ILAE, International League Against Epilepsy; *MECP2*, methyl-CpG binding protein 2; MEG, magnetoencephalography; MRI, magnetic resonance imaging; NDD, neurodevelopmental disorder; NGS, next-generation sequencing; NODDI, neurite orientation dispersion and density imaging; *SCN1A*, sodium voltage-gated channel alpha subunit 1; SEEG, stereoelectroencephalography; TLE, temporal lobe epilepsy; VBM, voxel-based morphometry; WES, whole exome sequencing; WGS, whole genome sequencing
